# Calmodulin-Dependent Regulation of Overexpressed but Not Endogenous TMEM16A Expressed in Airway Epithelial Cells

**DOI:** 10.3390/membranes11090723

**Published:** 2021-09-21

**Authors:** Khaoula Talbi, Jiraporn Ousingsawat, Raquel Centeio, Rainer Schreiber, Karl Kunzelmann

**Affiliations:** Physiological Institute, University of Regensburg, University Street 31, D-93053 Regensburg, Germany; khaoula.talbi@vkl.uni-regensburg.de (K.T.); jiraporn.ousingsawat@vkl.uni-regensburg.de (J.O.); raquel.centeio@vkl.uni-regensburg.de (R.C.); rainer.schreiber@vkl.uni-regensburg.de (R.S.)

**Keywords:** TMEM16A, calmodulin, CAMKII, calcineurin, Ca^2+^-activated Cl^−^ channel, anoctamin 1, CAM

## Abstract

Regulation of the Ca^2+^-activated Cl^−^ channel TMEM16A by Ca^2+^/calmodulin (CAM) is discussed controversially. In the present study, we compared regulation of TMEM16A by Ca^2+^/calmodulin (holo-CAM), CAM-dependent kinase (CAMKII), and CAM-dependent phosphatase calcineurin in TMEM16A-overexpressing HEK293 cells and TMEM16A expressed endogenously in airway and colonic epithelial cells. The activator of the Ca^2+^/CAM-regulated K^+^ channel KCNN4, 1-EBIO, activated TMEM16A in overexpressing cells, but not in cells with endogenous expression of TMEM16A. Evidence is provided that CAM-interaction with TMEM16A modulates the Ca^2+^ sensitivity of the Cl^−^ channel. Enhanced Ca^2+^ sensitivity of overexpressed TMEM16A explains its activity at basal (non-elevated) intracellular Ca^2+^ levels. The present results correspond well to a recent report that demonstrates a Ca^2+^-unbound form of CAM (apo-CAM) that is pre-associated with TMEM16A and mediates a Ca^2+^-dependent sensitization of activation (and inactivation). However, when using activators or inhibitors for holo-CAM, CAMKII, or calcineurin, we were unable to detect a significant impact of CAM, and limit evidence for regulation by CAM-dependent regulatory proteins on receptor-mediated activation of endogenous TMEM16A in airway or colonic epithelial cells. We propose that regulatory properties of TMEM16A and and other members of the TMEM16 family as detected in overexpression studies, should be validated for endogenous TMEM16A and physiological stimuli such as activation of phospholipase C (PLC)-coupled receptors.

## 1. Introduction

In our earlier study we analyzed the regulation of the Ca^2+^-activated Cl^−^ channel (CaCC) TMEM16A. We found that the channel can be activated by activators of Ca^2+^/calmodulin (CAM)-regulated KCNN4 K^+^ channels, such as 1-EBIO and riluzole [1]. In addition, we demonstrated that TMEM16A physically interacts with CAM. A subsequent report indicated that the anion selectivity of TMEM16A is dynamically regulated by the Ca^2+^/CAM complex [2]. This study showed that CAM reversibly increases the permeability ratio P_HCO3-_/P_Cl_^−^. Further support for a CAM-dependent regulation of TMEM16A came from an interesting study by the Colecraft team [3]. They described a Ca^2+^-unbound form of CAM (apo-CAM), which is pre-associated with TMEM16A or the paralogue TMEM16B channel complexes. Apo-CAM mediates a Ca^2+^ dependent sensitization of activation and Ca^2+^ dependent inactivation of TMEM16 channels [3,4]. Moreover, CAM-dependent activation and inactivation of TMEM16A had also been found in an earlier study [5]. In contrast to these studies, other laboratories did not find evidence for regulation of TMEM16A by CAM or alteration of the bicarbonate permeability of TMEM16A by CAM [6,7].

While direct a CAM-regulation of TMEM16A is discussed controversially, several studies demonstrate that CAM-dependent kinase II (CAMKII) regulates CaCC/TMEM16A. However, CAMKII was found to activate [8,9,10,11,12] and to inhibit [13,14,15,16,17] CaCC and TMEM16A, respectively. Greenwood and Leblanc found a cell-type-dependent activation/inhibition of CaCC by CAMKII [18], while Ko et al. found differential regulation of TMEM16A by CAMKII, depending on the splice variant [19,20]. One study showed modulation of TMEM16A Cl^−^ currents by CaMKIIγ phosphorylation at serine residues in TMEM16A. Serine525 and Serine727 in TMEM16A were mutated to alanine, but only mutation at Ser727 (S727A) reversed the CaMKIIγ inhibition of the TMEM16A Cl^−^ current [15].

Earlier studies describe a crosstalk between Ca^2+^/CAMKII-dependent regulation and inositol (3,4,5,6)-phosphate dependent regulation of CaCC, which also appears to be cell-type-dependent, possibly explaining some of the controversial findings outlined above [21,22]. In our earlier study, we observed that regulation of TMEM16A by INO-4995, a lipid inositol phosphate, differs depending on whether the channel is overexpressed in mammalian cells such as HEK293, or whether regulation of endogenous TMEM16A is examined [23]. While overexpressed TMEM16A was potently activated by the inositol phosphate INO-4995, endogenous TMEM16A expressed in airway and colonic epithelial cells was not. Activation of endogenous TMEM16A by purinergic receptor stimulation was augmented by INO-4995 [23]. Along the same line, the membrane permeable dioctanoyl-PIP_2_ phosphatidylinositol 4,5-bisphosphate-diC8 activated overexpressed TMEM16A, but did not touch endogenous TMEM16A [24]. Furthermore, other previously reported activators of TMEM16A such as melittin or cinnamaldehyde failed to activate endogenous TMEM16A. These differences prompted us to compare the role of CAM for acute receptor-mediated activation of TMEM16A in overexpressing cells and epithelial cells with endogenous expression of TMEM16A. Here, we made use of four different cell lines. HEK293 cells were used to study regulation of overexpressed TMEM16A, while airway epithelial cells (BCi_NS1, CFBE) and colonic HT_29_ cells were used to study regulation of endogenous TMEM16A. We demonstrate the absence of a significant CAM-dependent regulation for endogenous TMEM16A, solidifying the conclusion that control by CAM and other regulatory properties are indeed different for endogenous and overexpressed TMEM16A. Moreover, we made use of independent techniques (patch clamp, Ussing chamber, YFP-quenching) to reduce the chance for misinterpretations due to methodological artefacts. The data suggest only a minor role of CAM and the CAM-dependent proteins CAMKII and calcineurin for activation of endogenous TMEM16A, while CAM is likely to enhance Ca^2+^ sensitivity of overexpressed TMEM16A.

## 2. Materials and Methods

### 2.1. Cell Culture

All cells were grown at 37 °C in a humidified atmosphere with 5% (v/v) CO_2_. Culture conditions for CFBE and HT_29_ cells have been described earlier [25]. In brief, airway epithelial cells were grown in DMEM/Ham’s F-12 with L-Glutamine medium supplemented with 10% (v/v) fetal bovine serum (FBS), 1% (v/v) L-glutamine 200mM and 1% (v/v) HEPES 1M (all from Capricorn Scientific, Ebsdorfergrund, Germany). CFBE parental cells were grown in MEM with Earle’s Salts with L-Glutamine medium (Capricorn Scientific, Ebsdorfergrund, Germany) supplemented with 10% FBS. The airway epithelial cell line H441 was grown in RPMI and DMEM media. The immortalized human airway basal cell line BCi-NS1 (kindly provided by Prof. Ron Crystal, Weill Cornell Medical College, New York City, NY, USA) was maintained in Bronchial Epithelial Growth Media (Lonza). Cells were differentiated by growing on permeable supports (Snapwell, Corning, NY, USA) in an air-liquid interface (ALI) for up to 30 days in PnemaCult-ALI medium supplemented with PneumaCult-ALI 10X Supplement, PneumaCult-ALI Maintenance Supplement, hydrocortisone and heparin (all from StemCell Technologies, Vancouver, BC, Canada), and 1% penicilin-steptomycin (10 000 U/mL; Gibco, ThermoFisherScientific, Waltham, MA, USA).

### 2.2. cDNA, siRNA-TMEM16A, RT-PCR

Construction of pcDNA31 human TMEM16A (abc) has been described previously [26]. Cells were transfected using standard protocols for Lipofectamine 3000. Knockdown of TMEM16A in CFBE parental cells was performed by transfecting siTMEM16A (5-CCUGUACGAAGAGUGGGCACGCUAU-3, Invitrogen, Carlsbad, CA, USA) using standard protocols for Lipofectamine 3000 (Invitrogen, Carlsbad, CA, USA). Scrambled siRNA (Silencer^®^ Select Negative Control siRNA #1, Ambion, Austin, TX, USA) was transfected as negative control. All experiments were performed 72 h after transfection. For RT-PCR total RNA from HT29 and CFBE cells were isolated using NucleoSpin RNA II columns (Macherey-Nagel, Düren, Germany). Total RNA (0.5 µg / 25 µL reaction) was reverse-transcribed using random primer (Promega, Mannheim, Germany) and M-MLV Reverse Transcriptase RNase H Minus (Promega, Mannheim, Germany). Each RT-PCR reaction contained sense (0.5 µM) and antisense primer (0.5 µM) (Table 1), 0.5 µL cDNA and GoTaq Polymerase (Promega, Mannheim, Germany). After 2 min at 95 °C, cDNA was amplified (targets 35 cycles, reference Gapdh 25 cycles) for 30 s at 95 °C, 30 s at 56 °C, and 1 min at 72 °C. PCR products were visualized by loading on Midori Green Xtra (Nippon Genetics Europe) containing agarose.

### 2.3. Patch Clamping

Cells were patch-clamped after growing on coated glass coverslips for 2–3 days. Whole cell patch clamp techniques and data analysis have been described earlier [27]. In brief, patch pipettes were filled with a cytosolic-like solution containing (in mM): KCl 30, K-Gluconate 95, NaH_2_PO_4_ 1.2, Na_2_HPO_4_ 4.8, EGTA 1, Ca-Gluconate 0.758, MgCl_2_ 1.03, D-Glucose 5, ATP 3; pH 7.2. The intracellular (pipette) Ca^2+^ activity was 0.1 µM. Fast whole cell current recordings were performed as described recently [28]. The bath was perfused continuously with standard bicarbonate-free Ringer’s solution (in mM: NaCl 145, KH_2_PO_4_ 0.4, K_2_HPO_4_·3 H_2_O 1.6, Glucose 5, MgCl_2_·6 H_2_O 1, Ca-Gluconate·1 H_2_O 1.3) at a rate of 8 mL/min. Patch pipettes had an input resistance of 2–4 MΩ and whole cell currents were corrected for serial resistance. Currents were recorded using a patch clamp amplifier (EPC 7, List Medical Electronics, Darmstadt, Germany), the LIH1600 interface, and PULSE software (HEKA, Lambrecht, Germany), as well as Chart software (AD Instruments, Spechbach, Germany). Cells were stimulated with 1, 10, or 100 µM ATP in standard bicarbonate-free Ringer’s solution. Cells were current-clamped for most of the time. In regular intervals, membrane voltage (*V*c) was clamped in steps of 20 mV from −100 to +100 mV. The inhibitor of Ca^2+^-activated KCNN4 K^+^ channels, if indicated TRAM-34 (100 nM), was present in the patch clamp and other experiments to avoid contributions of Ca^2+^-activated K^+^ channels.

### 2.4. Ussing Chamber

Short circuit Ussing chamber measurements were performed on permeable support-grown BCi-NS1 cells. Experiments were performed under short circuit Ussing chamber conditions (Physiological instruments) in the presence of 5% CO_2_ and 25 mM HCO_3_^−^ at 37 °C, as described earlier [29].

### 2.5. YFP Quenching

Cells stably transfected with iodide-sensitive YFP were plated in transparent 96-well plates (Sarstedt, Nümbrecht, Germany), cultured 24–72 h to 80–90% confluence, washed with gluconate substituted-Ringer solution (mmol/L: NaCl 120; Na^+^-gluconate 20; KCl 5; MgCl2 1; CaCl2 2; D-Glucose 10; HEPES 10), and incubated with or without test compounds in this solution. Total intracellular YFP fluorescence intensity in each well was measured in a fluorescence microplate reader (NOVOstar, BMG Labtech, Ortenberg, Germany) kept at 37 °C, using an excitation wavelength of 485 nm and emission detection at 520 nm. Fluorescence was read continuously during injection of an iodide (I−)-substituted Ringer solution (mmol/L: NaCl 120; NaI 20; KCl 5; MgCl2 1; CaCl2 2; D-Glucose 10; HEPES 10) by a syringe pump and following injections of a symmetrical Ringer solution (in mmol/L: NaCl 100–120; Na^+^-Gluconate 10–20; NaI 10–20; KCl 5; MgCl2 1; CaCl2 2; D-Glucose 10; HEPES 10) carrying test compounds. Original data were collected, background fluorescence was subtracted, and the initial rate of maximal fluorescence decay caused by I- influx/YFP fluorescence-quenching upon acute injection (or pre-incubation) of test compounds was measured to determine anion conductance/activity of TMEM16A.

### 2.6. Materials and Statistical Analysis

All compounds used were of highest available grade of purity and were obtained from Sigma-Aldrich (St. Louis, Missouri, USA), unless indicated otherwise. Data are shown as individual traces/representative images and/or as summaries with mean values ± SEM, with the respective number of experiments given in each figure’s legend. The population-effective sizes for the experiments were typically around 0.87–0.94. As we did not want to rely on results obtained in one cell line only, we compared results obtained in overexpressing HEK293 cells with those obtained in cell expressing endogenous TMEM16A channels (CFBE, BCi-NS1, HT_29_ cells). For statistical analysis, paired or unpaired Student’s *t*-test or ANOVA were used where appropriate. A *p*-value of < 0.05 was accepted as a statistically significant difference.

## 3. Results

### 3.1. Overexpressed TMEM16A Is Spontaneously Active Due to Enhanced Ca^2+^ Sensitivity

**Figure 1:**
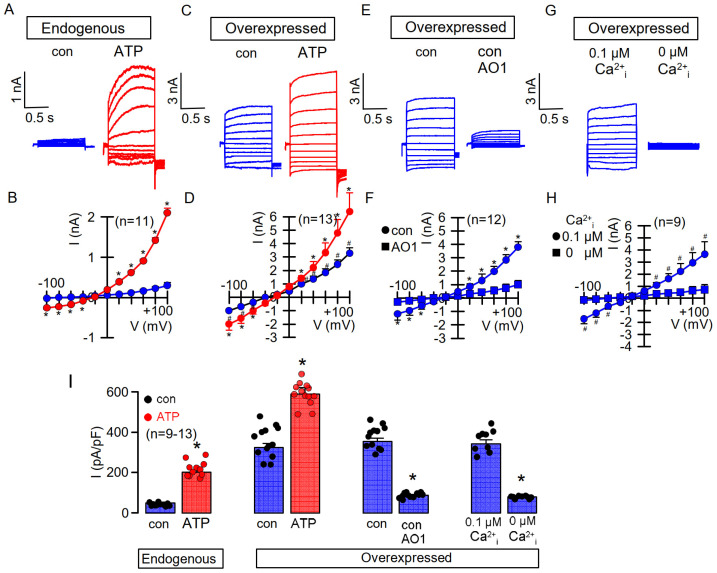
Overexpressed TMEM16A is spontaneously active due to enhanced Ca^2+^ sensitivity. TMEM16A whole cell currents were activated by 100 µM ATP in CFBE airway epithelial cells expressing TMEM16A endogenously (**A**,**B**) and in HEK293 cells overexpressing TMEM16A (**C**,**D**). **A**,**B**) TMEM16A is closed under control conditions ([Ca^2+^]_i_ = 0.1 µM) and is opened by ATP-induced increase in [Ca^2+^]_i_.(**C,D**) Whole cell currents were detected in TMEM16A-overexpressing cells under control conditions, with additional activation by ATP. (**E**,**F**) Whole cell currents detected under basal conditions were inhibited by the TMEM16A-blocker CaCCinhAO1 (AO1, 20 µM). (**G**,**H**) Whole cell currents detected under control conditions ([Ca^2+^]_i_ = 0.1 µM) were not detected in the absence of Ca^2+^ in the pipette filling solution. (**I**) Summary of the current densities. Mean ± SEM (n = number of experiments). * Significant activation by ATP and inhibition by AO1, respectively (*p* < 0.05; paired *t*-test). ^#^ Significant difference when compared to endogenous currents or in presence of 0 µM [Ca^2+^]_i_, respectively (*p* < 0.05; unpaired *t*-test).

We observed in earlier studies that TMEM16A, when overexpressed in HEK293 cells, produces whole cell currents in the presence of basal cytosolic Ca^2+^ concentrations of 0.1 µM and with physiological concentrations of K^+^, Na^+^, and Cl^−^ in the patch pipette filling solution [30,31] (Figure 1C,D). Such basal currents were not observed in cells expressing TMEM16A endogenously, such as HT_29_ colonic epithelial or CFBE airway epithelial cells [31] (Figure 1A,B). Here, TMEM16A was activated only after increase in the intracellular Ca^2+^ concentration, e.g., by application of the purinergic agonist ATP [31] (Figure 1A,B). In overexpressing cells, application of ATP activated whole cell currents in addition to the already enhanced basal currents. Enhanced and activated currents were inhibited by different blockers of TMEM16A such as CaCCinhAO1 (AO1) or niflumic acid [1,30]. Moreover, enhanced basal currents were inhibited by application of AO1 or in the absence of pipette (cytosolic) Ca^2+^, indicating that TMEM16A is in charge of both enhanced basal and ATP-activated currents (Figure 1E–H). These data strongly suggest a higher Ca^2+^ sensitivity of overexpressed TMEM16A when compared with endogenous TMEM16A.

**Figure 2:**
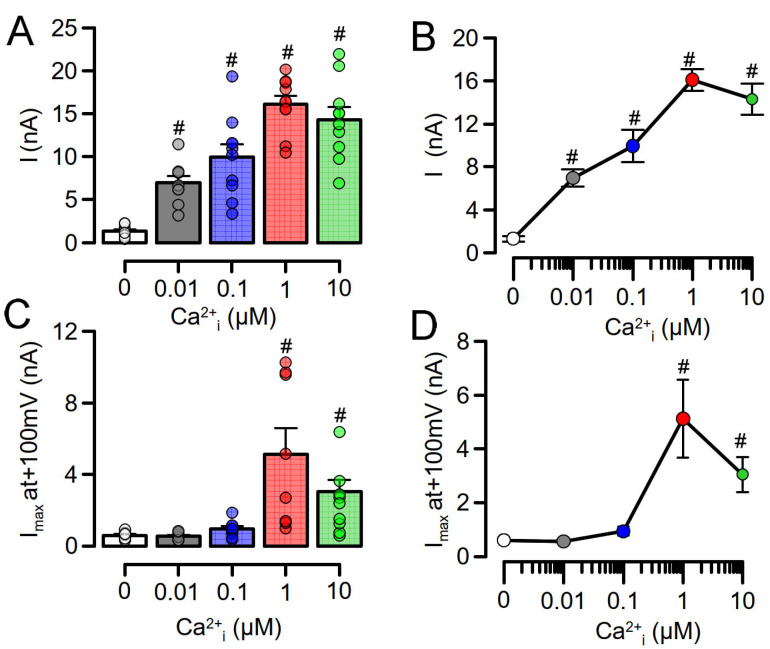
Enhanced Ca^2+^ sensitivity of overexpressed TMEM16A. Patch pipettes were filled with solutions containing different free Ca^2+^ concentrations (0–10 µM). At 0 µM pipette Ca^2+^ no TMEM16A, whole cell currents were observed in overexpressing HEK293 cells or CFBE cells expressing endogenous TMEM16A. (**A**,**B**) Whole cell currents were activated in overexpressing HEK293 cells at Ca^2+^ concentrations as low as 0.01 µM. (**C**,**D**) TMEM16A currents in CFBE cells were only observed at 1 µM Ca^2+^. Mean ± SEM (n = number of experiments). ^#^ Significant activation when compared to 0 µM Ca^2+^ (*p* < 0.05; ANOVA).

To further demonstrate enhanced Ca^2+^ sensitivity of overexpressed TMEM16A, we performed patch clamp experiments in which we loaded the patch pipette with pipette filling solution containing different free Ca^2+^ concentrations. At 0 µM pipette Ca^2+^ no TMEM16A, whole cell currents were observed in overexpressing HEK293 cells or CFBE cells expressing endogenous TMEM16A. Whole cell currents were activated in overexpressing HEK293 cells at Ca^2+^ concentrations as low as 0.01 µM (10^−8^ M), while TMEM16A currents in CFBE cells were only observed at 1 µM (10^−6^ M) Ca^2+^, again strongly suggesting a higher Ca^2+^ sensitivity for overexpressed TMEM16A (Figure 2). At 10 µM Ca^2+^, whole cell currents were smaller due to the well-known Ca^2+^-dependent inactivation of TMEM16A [23]. No whole cell currents were activated in mock-transfected HEK293 cells.

### 3.2. Enhanced Ca^2+^ Sensitivity of Overexpressed TMEM16A Is Modulated by CAM

**Figure 3:**
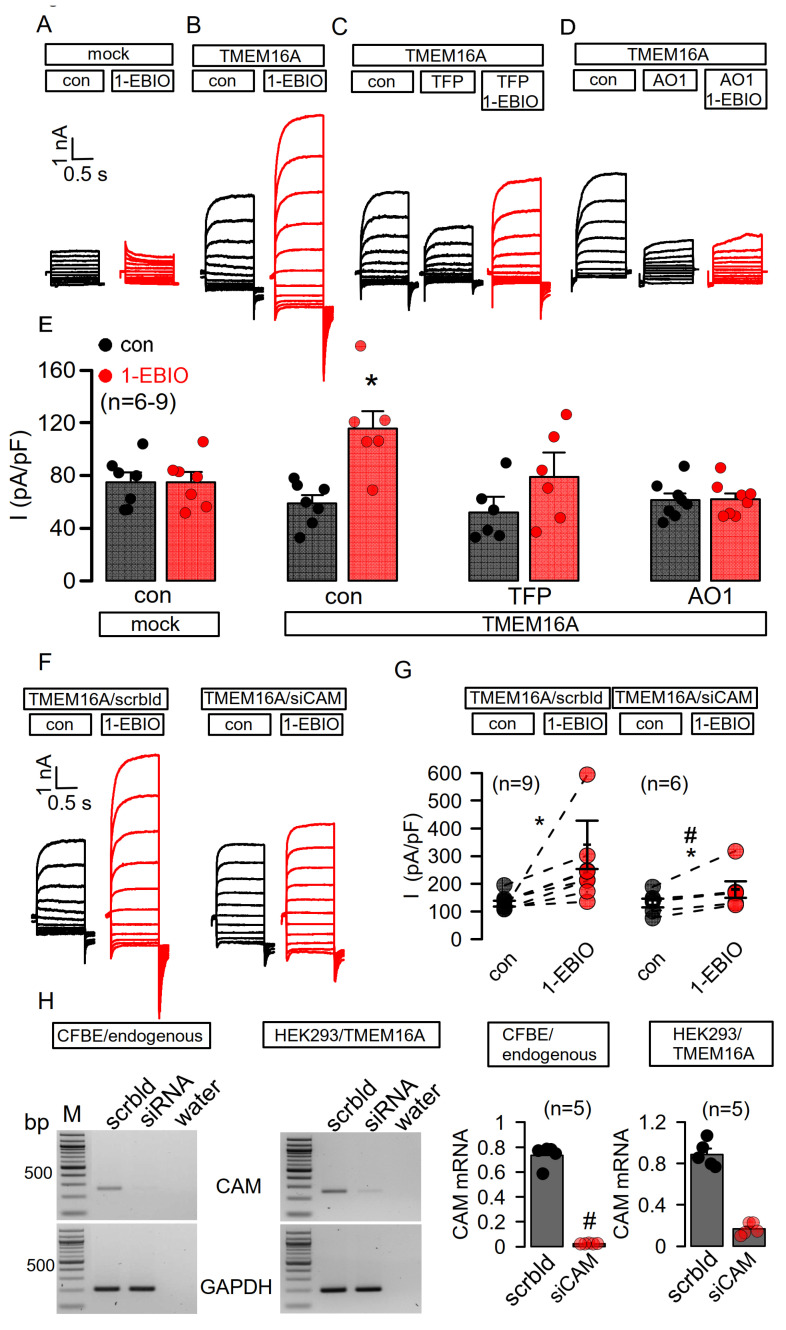
Enhanced Ca^2+^ sensitivity of overexpressed TMEM16A is mediated by CAM. (**A**–**D**) No effect of 1-EBIO (1 mM) on whole cell currents in mock-transfected HEK293 cells. Basal activity of TMEM16A and further activation by 1-EBIO in TMEM16A-overexpressing HEK293 cells. Inhibition of basal TMEM16A-activity and attenuation of the effect of 1-EBIO by the CAM-inhibitor trifluoperazine (TFP, 10 µM). Inhibition of basal TMEM16A-activity and inhibition of TMEM16A-activation by 1-EBIO using CaCC-inhAO1 (AO1, 20 µM). (**E**) Summary of the current densities. (**F**,**G**) Basal activity of TMEM16A and further activation by 1-EBIO in overexpressing HEK cells treated with scrambled RNA (scrbld) or with siRNA for CAM (siCAM). (**H**) Knockdown of CAM was assessed by semiquantitative RT-PCR in both CFBE cells expressing endogenous TMEM16A and in HEK293 cells overexpressing TMEM16A. Similar number of CAM-transcripts are expressed in CFBE and HEK293 cells. Mean ± SEM (n = number of experiments). * Significant activation by 1-EBIO and inhibition by AO1 or TFP, respectively (*p* < 0.05; paired *t*-test). ^#^ Significant difference when compared to activation in the absence of AO1, TFP, or siCAM, respectively (*p* < 0.05; unpaired *t*-test).

We previously reported regulation of TMEM16A by CAM [1], which, however, has been discussed controversially (c.f. Introduction). Here, we examined activation of overexpressed TMEM16A by the activator of the CAM/KCNN4 complex, 1-EBIO. No effects were seen for 1-EBIO in mock-transfected HEK293 cells. In contrast, TMEM16A-overexpressing cells showed enhanced basal currents, which were further augmented by 1-EBIO, clearly suggesting CAM-regulation of TMEM16A. Application of the CAM-inhibitor trifluoperazine (TFP) inhibited enhanced basal currents and attenuated activation by 1-EBIO. Moreover, the TMEM16A-inhibitor CaCCinhAO1 (AO1) [32] strongly inhibited enhanced basal currents and abolished the effect of 1-EBIO (Figure 3A–E). Finally, activation by 1-EBIO was examined in overexpressing HEK293 cells treated with scrambled RNA or with siRNA for CAM. Activation by 1-EBIO was attenuated in cells in which expression of CAM was knocked down (Figure 3F,G). Knockdown of CAM was assessed by semiquantitative RT-PCR and was 94 ± 2 % (n = 5). A similar number of CAM-transcripts are expressed in CFBE and HEK293 cells (Figure 3H). Notably, in contrast to TMEM16A-overexpressing HEK293 cells, no effect of siRNA-CAM was observed on the activation of TMEM16A in CFBE cells. These results suggest that enhanced basal TMEM16A currents are due to enhanced Ca^2+^ sensitivity, probably modulated by association of CAM with TMEM16A.

### 3.3. No Activation of Endogenous TMEM16A by 1-EBIO and No CAMKII-Dependent Regulation of TMEM16A in Airway Epithelial Cells

**Figure 4:**
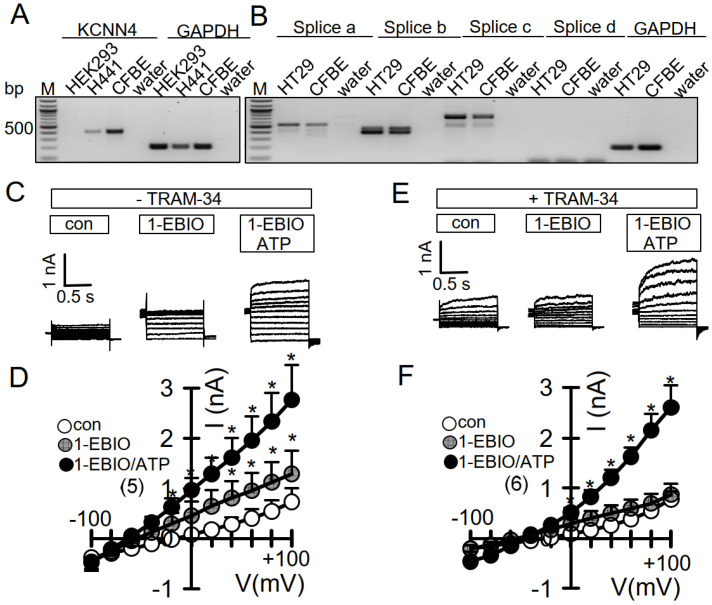
No activation of endogenous TMEM16A by 1-EBIO in CFBE airway epithelial cells. *(***A**) RT-PCR analysis indicating expression of Ca^2+^-activated KCNN4 channels in H441 and CFBE airway epithelial cells but not in HEK293 cells. (**B**) RT-PCR analysis indicating expression of the splice variant TMEM16Aa,b,c in HT_29_ colonic epithelial and CFBE airway epithelial cells. (**C**,**D**) Whole cell currents and current/voltage relationships demonstrating current activation and hyperpolarization by 1-EBIO (1 mM) and additional activation by ATP (100 µM). (**E**,**F**) Activation of whole cell currents and hyperpolarization by 1-EBIO is inhibited by the KCNN4 blocker TRAM-34 (100 nM). Mean ± SEM (n = number of experiments). * Significant activation by 1-EBIO and additional activation by ATP (*p* < 0.05; paired *t*-test).

We examined whether CAM-dependent regulation can also be detected for TMEM16A expressed endogenously in CFBE airway epithelial cells. Expression of TMEM16A in CFBE cells has been shown previously [25,27]. We also found expression of Ca^2+^-activated KCNN4 channels in CFBE (and H441) airway epithelial cells, but not in HEK293 cells (Figure 4A). Both CFBE and HT_29_ colon epithelial cells express the splice variant TMEM16Aa,b,c (Figure 4B). Patch clamp experiments were performed in the absence or presence of TRAM-34, a potent inhibitor of Ca^2+^-activated KCNN4 K^+^ channels. In the absence of TRAM-34, 1-EBIO activated a whole cell current and hyperpolarized the membrane voltage (Figure 4C,D). In contrast, in the presence of TRAM-34, no hyperpolarization was observed, and 1-EBIO did not activate whole cell currents (Figure 4E,F). Under both conditions, ATP activated whole cell currents. The data suggest that in airway epithelial cells with endogenous TMEM16A, 1-EBIO activates KCNN4 but not TMEM16A.

**Figure 5:**
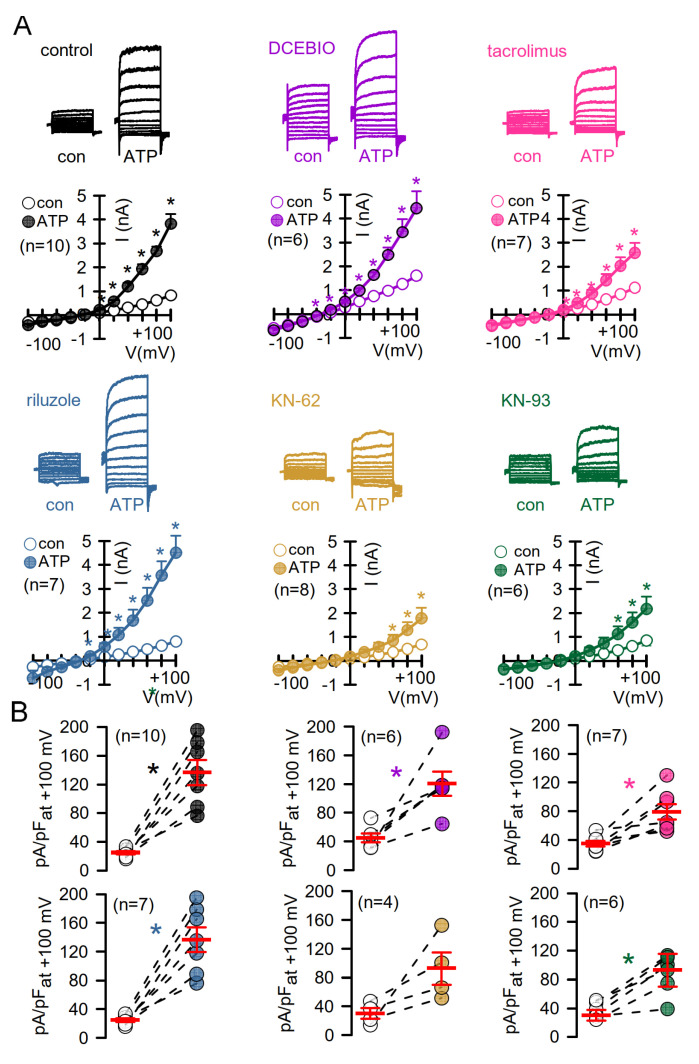
Evidence for CAMKII-dependent activation of TMEM16A but not for CAM- or calcineurin-dependent regulation of endogenous TMEM16A in CFBE airway epithelial cells. (**A**) Whole cell patch clamp analysis of ATP (100 µM)-activated TMEM16A currents and effects of the 1-EBIO analogues DCEBIO and riluzole (both 10 µM), the CAMKII-inhibitors KN-62 and KN-93 (both 10 µM) and the calcineurin inhibitor tacrolimus (10 µM). Cells were pre-incubated with the compounds, which were also present during the experiment. Under all conditions, significant activation of whole cell currents by ATP was observed. While the KCNN4 activators DCEBIO and riluzole enhanced ATP-activated currents, KN-62, KN-93, and tacrolimus reduced current activation. The time course for ATP-activation of TMEM16A remained unchanged by the drugs. (**B**) Calculated current densities. All experiments were performed in the presence of the KCNN4-inhibitor TRAM-34. Mean ± SEM (n = number of experiments). * Significant activation by ATP (*p* < 0.05; paired *t*-test).

Because CAM-dependent kinase (CAMKII) and the serine/threonine-phosphatase calcineurin require CAM to operate, we examined the effects of other CAM-activators (DCEBIO, riluzole), CAMKII-inhibitors (KN-62, KN-93), and the calcineurin-inhibitor tacrolimus (FK-506) on basal and ATP-activated endogenous currents in airway epithelial cells. Notably, DCEBIO enhanced basal currents, and both DCEBIO and riluzole somewhat augmented ATP-activated currents but failed to reach significance (Figure 5A,B). Tacrolimus and inhibition of CAMKII attenuated ATP-activated currents. Taken together, apart from CAMKII-dependent activation, there is no clear evidence for CAM or calcineurin-dependent regulation of endogenous TMEM16A in CFBE airway epithelial cells.

### 3.4. No Evidence for CAM, CAMKII, or Calcineurin-Dependent Regulation of Endogenous TMEM16A Expressed in Bci-NS1 Airway Epithelial Cells

**Figure 6:**
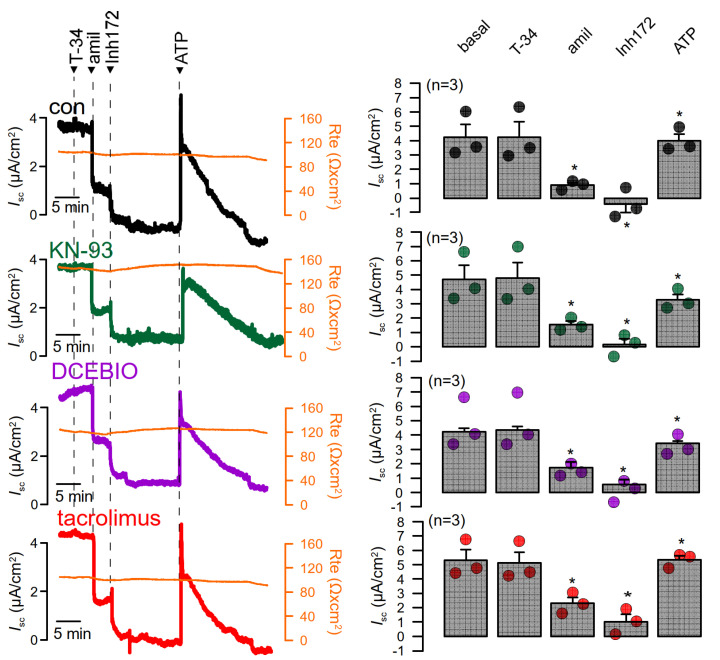
No evidence for CAM, CAMKII, or calcineurin-dependent regulation of endogenous TMEM16A in BCi-NS1 airway epithelial cells. Left panels: Ussing chamber recordings from human BCi-NS1 airway epithelial cells, assessing the ion transport by continuously measuring the transepithelial short circuit current Isc. Original continuous recordings of the short circuit currents Isc in the absence or presence of different inhibitors. The transepithelial resistance was continuously monitored in parallel. Cells were measured under control conditions or after exposure to KN-93 (10 µM), DCEBIO (10 µM), or tacrolimus (100 nM). Right panels: Summaries for the short circuit currents corresponding to continuous Isc recordings shown in the left panels. The effects of TRAM-34 (T-34, 100 nM), amiloride (amil, 10 µM), CFTRinh172 (Inh172, 20 µM), and ATP (5 µM) on Isc are summarized. Mean ± SEM (n = number of experiments). * Significant effects of amil, Inh172, and ATP (*p* < 0.05; paired *t*-test).

In order to exclude cell-specific or methodological factors, we re-examined CAM, CAMKII, or calcineurin-dependent regulation in Ussing chamber recordings with human BCi-NS1 airway epithelial cells. When grown on permeable supports, these cells differentiate into a polarized epithelium with airway epithelial typical properties [25,33]. Cells were measured under control conditions or after exposure to KN-93, DCEBIO, or tacrolimus. Compounds were added subsequently to luminal or basolateral sides of the epithelium, and the effects of TRAM-34 (T-34), the epithelial Na^+^ channel inhibitor amiloride (amil), the inhibitor of the cystic fibrosis transmembrane conductance regulator Cl^−^ channel CFTRinh172 (Inh172), and ATP on short circuit currents (Isc) were examined. Neither Na^+^ absorption by ENaC nor basal Cl^−^ currents by CFTR or ATP-activated TMEM16A/CFTR currents were significantly affected by any of the inhibitors (Figure 6).

### 3.5. No Evidence for CAM, CAMKII, or Calcineurin-Dependent Regulation of Endogenous TMEM16A in HT_29_ Colonic Epithelial Cells

**Figure 7:**
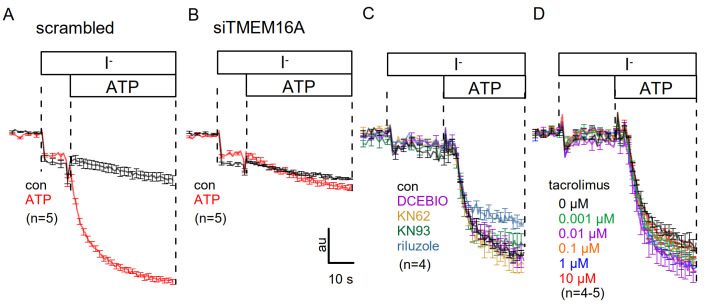
*No evidence for CAM, CAMKII, or calcineurin-dependent regulation of endogenous TMEM16A in HT_29_ colonic epithelial cells.* Iodide quenching of YFP stably expressed in HT_29_ colonic epithelial cells was used to assess 5µM ATP-activated halide permeability by activation of TMEM16A. (**A**) ATP-induced quenching in mock transfected HT_29_ cells. (**B**) ATP-induced quenching in HT_29_ cells with siRNA-knockdown of TMEM16A. (**C**) Preincubation of the cells with DCEBIO, KN-62, KN-93, and riluzole (all 10 µM) did not affect ATP-induced quenching. (**D**) Incubation with different concentrations of tacrolimus did not affect ATP-induced quenching. All experiments were performed in the presence of TRAM-34. Mean ± SEM (n = number of experiments). au = arbitrary units for fluorescence quenching.

We finally examined if any of the inhibitors affect ATP-activated TMEM16A whole cell currents in HT_29_ colonic epithelial cells. Here, we used iodide quenching of halide-sensitive yellow-fluorescent protein (YFP), stably expressed in HT_29_ cells. ATP-induced quenching in HT_29_ cells transfected with scrambled RNA was impressive (Figure 7A) and was completely abolished by siRNA-knockdown of TMEM16A (Figure 7B), indicating that TMEM16A was fully in charge of ATP, i.e., Ca^2+^-activated whole cell currents in HT_29_ cells. None of the inhibitors DCEBIO, KN-62, KN-93, riluzole, or tacrolimus, applied at different concentrations, affected current activation by ATP (Figure 7C,D). Taken together, the results suggest that CAM-dependent regulation of TMEM16A is present in overexpressing HEK293 cells but shows little effects on TMEM16A expressed endogenously in airway and colonic epithelial cells.

## 4. Discussion

Regulation of the Ca^2+^-activated Cl^−^ channel TMEM16A has been studied extensively. In most patch clamp studies, the channel is examined as overexpressed protein activated by high pipette Ca^2+^ in the presence of unphysiological salt concentrations and at 20 °C [34,35]. Our previous work showed that activation of TMEM16A by phosphatidylinositol 4,5-bisphosphate or inositol 3,4,5,6-tetrakisphosphate is well detected for overexpressed TMEM16A, but not for endogenous TMEM16A [23,24]. Moreover, other activators or inhibitors also show differential effects between overexpressed and endogenous channels [24]. The overexpressed channel is already active at basal intracellular (pipette) Ca^2+^ as low as 0.01 µM. This enhanced basal current is reduced at temperatures lower than 37 °C [30,31]. Notably, similar is observed for TMEM16F, which is a phospholipid scramblase and a nonselective ion channel [31]. Interestingly, the calcium-hypersensitive aspartic acid 408-to-glycine 408 (D408G) TMEM16F mutant showed spontaneous scrambling and ion channel activity when overexpressed in HEK293 cells, but both scrambling and ion currents were not enhanced in macrophages isolated from knockin-mice expressing D408G-TMEM16F instead of wt-TMEM16F [31,36] (data not shown). For both TMEM16A and TMEM16F, we proposed a role of lipid-dependent regulation modulating their Ca^2+^ sensitivity and activity at basal Ca^2+^ activity. Lipid-dependent regulation may depend on the ambient temperature and other factors such as temperature-sensitive phospholipase A2, reactive oxygen species, and the cytosolic (pipette) ion composition [31,37]. Moreover, we speculated earlier that i) massively overexpressed TMEM16A may end up in non-raft compartments, which are more accessible to other cellular Ca^2+^ sources, ii) overexpressed TMEM16A may translocate its activating Ca^2+^ source, i.e., the endoplasmic reticulum, by membrane tethering, and iii) unknown antagonistic accessory proteins may be stoichiometrically underrepresented in TMEM16A-overexpressing cells [23,24,38,39]. It is also entirely possible that temperature-sensitive Ca^2+^-permeable transient receptor potential (TRP) channels are involved in the temperature-sensitive activation of TMEM16A [40,41,42]. Notably, TRP channels have also been shown to be regulated by PIP_2_ and CAM [43,44]. Alternatively, other accessory proteins may interact differentially under overexpressed or endogenous conditions [24].

Pre-association of Ca^2+^-free CAM (apoCAM) may preferentially occur with overexpressed TMEM16A and thus sensitize the channel for intracellular Ca^2+^ [3,4]. Accordingly, the benzimidazolone 1-EBIO may then promote the association between apoCAM and TMEM16A. 1-EBIO is a known activator of Ca^2+^ sensitive SK-channels [45,46]. SK channels, e.g., KCNN4, consist of a α-subunit that requires CAM as ß-subunit to gate the channel upon binding of Ca^2+^ to CAM. 1-EBIO enhances the interaction between the α-subunit and CAM and thereby shifts the [Ca^2+^]_i_/channel activity-relationship to lower [Ca^2+^]_i_ [47]. Notably, 1-EBIO did not activate any SK currents in the absence of [Ca^2+^]_i_ [47], and similarly, TMEM16A was not spontaneously active and was not activated by 1-EBIO in the absence of cytosolic (pipette) Ca^2+^ (Figure 1).

Association of a ß-subunit such as holoCAM or apoCAM with TMEM16A may not be surprising, given the recent findings of an association of the KCNQ1-K^+^ channel ß-subunit KCNE1 with TMEM16A [48]. Nevertheless, activation of TMEM16A by benzimidazolone compounds and the effects of CAM-regulated CAMKII or calcineurin were very moderate in epithelial cells with endogenous expression of TMEM16A. Taken together, it appears indispensable to examine whether activation by CAM and other accessory proteins/ß-subunits [48], phosphatidylinositols, temperature or small molecule activators, respectively, does exist for endogenous channels expressed in non-cultured cells and tissues under physiological conditions.

## Figures and Tables

**Table 1:** Primers for PCR.

Gene Accession Number	Primer	Size (bp)
TMEM16A (abcd) NM_001378092.1		
Splice variant a	s: 5′-GGACCCTGATGCCGAGTGC as: 5′-GGAGAAGGGATAGGAGAGTC	547
Splice variant b	s: 5′-ACAGCAAAACCCGGAGC as: 5′-TCTCTGGTCACACATCTCC	462
Splice variant c	s: 5′-ACAGCAAAACCCGGAGC as: 5′-GGATGATCCTTGACAGCCTC	703
Splice variant d	s: 5′-GAAGAAAGAGTCCAGAAAC as: 5′-CCGATCTCTCCATGTCAGC	136
KCNN4 NM_002250.3	s: 5′-GATTTAGGGGCGCCGCTGAC as: 5′-CTTGCCCCACATGGTGCCC	405
Gapdh NM_001289726	s: 5′-GTATTGGGCGCCTGGTCAC as: 5′-CTCCTGGAAGATGGTGATGG	200
